# Klf10 is involved in extracellular matrix calcification of chondrocytes alleviating chondrocyte senescence

**DOI:** 10.1186/s12967-023-04666-7

**Published:** 2024-01-13

**Authors:** Rong Peng, Jie Shang, Ning Jiang, Hsu Chi-Jen, Yu Gu, Baizhou Xing, Renan Hu, Biao Wu, Dawei Wang, Xianghe Xu, Huading Lu

**Affiliations:** 1 Department of Orthopedics, The Fifth Affiliated Hospital of Sun Yat-sen University, Zhuhai, 519000 Guangdong China; 2 Guangdong Provincial Key Laboratory of Biomedical Imaging, The Fifth Affiliated Hospital, Sun Yat-sen University, Zhuhai, 519000 Guangdong China; 3 Department of Orthopedics, The Affiliated Yantai Yuhuangding Hospital of Qingdao University, Yantai, 26400 Shandong China

## Abstract

Osteoarthritis (OA) is a chronic degenerative disease resulting joint disability and pain. Accumulating evidences suggest that chondrocyte extracellular matrix calcification plays an important role in the development of OA. Here, we showed that Krüppel-like factor 10 (Klf10) was involved in the regulation of chondrocyte extracellular matrix calcification by regulating the expression of Frizzled9. Knockdown of Klf10 attenuated TBHP induced calcification and reduced calcium content in chondrocytes. Restoring extracellular matrix calcification of chondrocytes could aggravate chondrocyte senescence. Destabilization of a medial meniscus (DMM) mouse model of OA, in vivo experiments revealed that knockdown Klf10 improved the calcification of articular cartilage and ameliorated articular cartilage degeneration. These findings suggested that knockdown Klf10 inhibited extracellular matrix calcification-related changes in chondrocytes and alleviated chondrocyte senescence.

**Supplementary Information:**

The online version contains supplementary material available at 10.1186/s12967-023-04666-7.

## Introduction

Osteoarthritis (OA) is a long-term chronic degenerative and disabling joint disease that is statistically the fourth leading cause of disability worldwide [1, 2]. The pathology of OA is characterized by progressive degeneration of articular cartilage, remodeling of subchondral bone, osteophyte formation, synovial inflammation, and calcification of joint structures such as cartilage, and the resulting joint dysfunction [3]. However, duo to complicated pathology, there is no useful way to treat OA [4].

Normally, calcification is integral to the formation and function of bone tissue. However, extracellular matrix calcification of cartilage and soft tissue occurs in OA [5]. Abnormal cartilage calcification is an important pathogenic process of OA, and is also strongly associated with the severity of OA. Thus, Calcium deposition in the cartilage matrix facilitates joint degeneration, and inhibiting extracellular matrix calcification in cartilage alleviates OA progress [6, 7]. Therefore, understanding the mechanisms on extracellular matrix calcification of cartilage is critical to identify new therapeutic targets to treat OA.

Krüppel-like factor 10 (Klf10) is an early target gene of the transforming growth factor-β/Smad signaling pathway and belongs to the Krüppel-like family of transcriptional regulators [8, 9]. Klf10 has a DNA-binding domain, containing three C2H2 zinc fingers, which can bind to the CACCC element or GC box in the promoter region [10, 11]. Klf10 plays an important role in bone biology, and its overexpression in chondrocytes inhibits cell proliferation and migration [12]. Knockout of Klf10 gene inhibits chondrocyte hypertrophy, which plays a key role in coordinating longitudinal bone growth and physiological cartilage calcification, resulting in skeletal dysplasia in mice [13, 14]. However, it is unknown that the role of klf10 in abnormal calcification in cartilage.

Frizzled9 (Fzd9) is a G-protein-coupled transmembrane receptor that is commonly expressed in brain, testis, skeletal muscle, and kidney tissue [15]. It is also the Wnt receptor of the Frizzled family, which positively regulates bone reconstruction through an atypical Wnt pathway without involving B-catenin-dependent signal transduction [16]. Fzd9 actively regulates intramembrane and intrachrondral bone formation during fracture healing [17].

Our previous studies have shown that Klf10 is highly expressed during OA, and intervention of its expression alleviated chondrocyte senescence and delay the OA progress [18]. However, the mechanisms regulating chondrocyte senescence remain unclear. Calcium alkaline phosphate and calcium pyrophosphate dihydrate, two main components in the pathological calcification crystallization, are related to the aging of chondrocytes, and calcium pyrophosphate dihydrate has a stronger effect on the aging of chondrocytes [19]. We hypothesized that Klf10 induced senescence of chondrocyte by abnormal calcification. In order to test our hypothesis, we found that Klf10 bound to the promoter of Fzd9 gene, and regulated its expression to affect the entry of calcium ions into cells. And knockdown Klf10 reduced extracellular matrix calcification in mouse primary chondrocytes. Restoring extracellular matrix calcification of chondrocytes with BCP or CPPD could aggravate chondrocyte senescence. In addition, knockdown of Klf10 in the medial meniscus (DMM) model of OA mice attenuated the extracellular matrix calcification of cartilage and improved cartilage degeneration.

## Results

### Extracellular matrix calcification occurs in senescence chondrocytes and OA cartilage

**Fig. 1:**
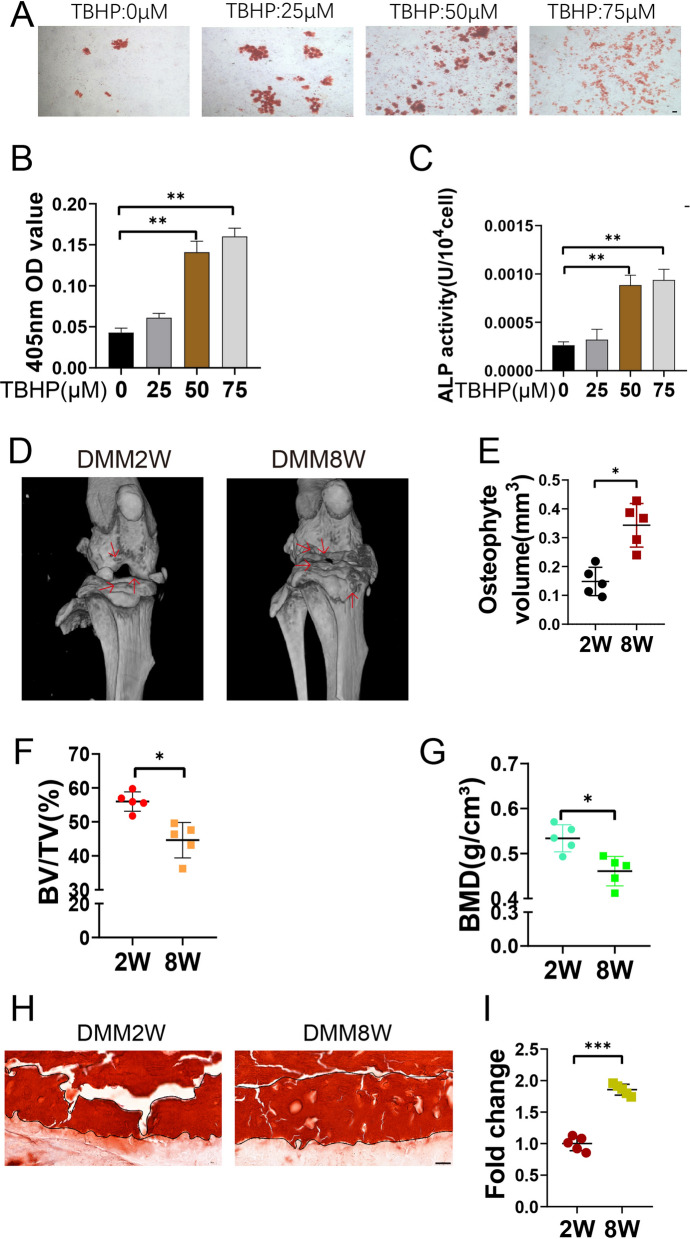
Extracellular matrix calcification occurs in senescence chondrocytes and OA cartilage. **A**, **B** Mouse articular chondrocytes were treated with different concentrations of TBHP, alizarin red dye was used to show the size of calcium nodules (n = 3). Scale bar: 100 μm. **C** The alkaline phosphatase kit was used to measure the alkaline phosphatase activity of chondrocytes treated with TBHP at different concentrations (n = 3). **D**–**G** Micro-CT was used to detect the knee joints of mice after DMM 2 and 8 weeks, and SKYscan software was used to analyze osteophyte volume, bone density and bone volume fraction (n = 5). **H**, **I** Alizarin red was used to stain the knee sections without decalcification. The black line area represented the calcified cartilage area (n = 5). Scale bar: 20 μm

Previously, we induced chondrocytes senescence by 1-Butyl Hydroperoxide, TBHP. In this research, we treated chondrocytes with different concentrations of TBHP and performed alizarin red staining to observe calcification (Fig. 1A). With increasing TBHP concentrations, the degree of chondrocyte calcification was aggravated. The calcification degree was the highest at 50 µM TBHP (Fig. 1B). Changes in ALP activity was also observed in cells treated with TBHP, and ALP was significantly also activated at 50 µM TBHP (Fig. 1C). These results suggested that chondrocyte calcification was related to senescence. We established a destabilized medial meniscus (DMM) mouse model, and micro-CT was used to analyze changes of joint parameters at different postoperative time points (Fig. 1D). With the extension of treatment time, the knee bone density and bone volume fraction in the 8-week DMM group significantly decreased compared that in the 2-week DMM group, but the osteophyte volume increased (Fig. 1E–G). Alizarin red staining showed that more calcified cartilage areas were formed in 8 weeks DMM model group (Fig. 1H, I). These results showed that extracellular matrix calcification occurred in senescence chondrocytes and OA cartilage, becoming progressively worse.

### Knockdown Klf10 attenuates TBHP induced chondrocytes extracellular matrix calcification

**Fig. 2:**
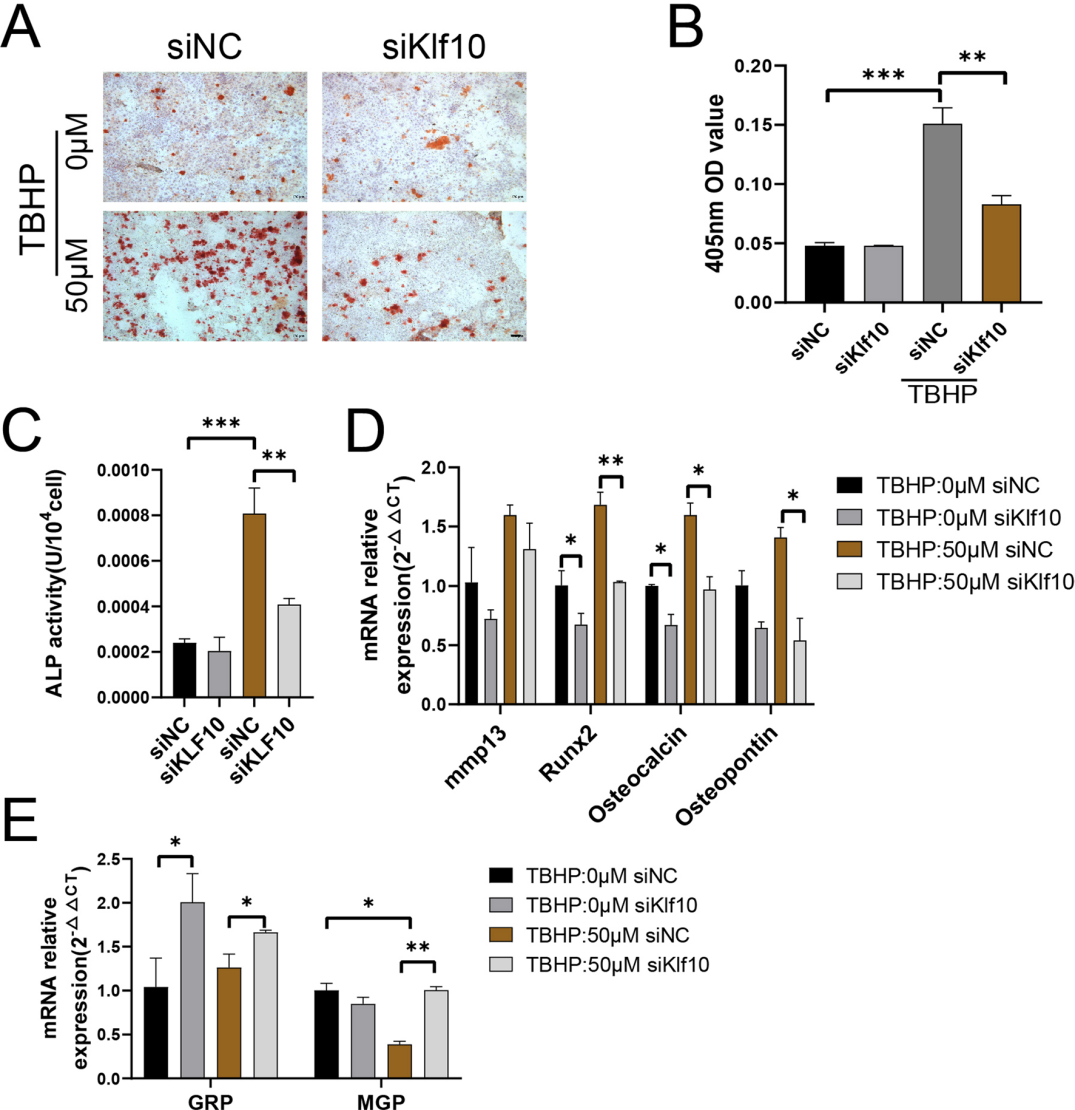
Klf10 knockdown attenuates TBHP induced chondrocytes extracellular matrix calcification. **A**, **B** Chondrocytes were treated with TBHP and siRNA (Klf10) and its negative control, and alizarin red staining was used to detect the formation of calcium nodules (n = 3). Scale bar: 100 μm. **C** The alkaline phosphatase kit was used to measure the alkaline phosphatase activity of chondrocytes (n = 3). **D**, **E** Relative calcification related gene mRNA expressions of murine chondrocytes were detected using RT-qPCR (n = 3)

Our previous research demonstrated that the expression of Klf10 was upregulated in OA chondrocytes, and Lee, J.M. et al. had demonstrated that Klf10 regulated hypertrophic differentiation of chondrocytes in growth plates [13]. To investigate whether Klf10 played a functional role in cartilage calcification, Klf10 siRNA was transfected to chondrocytes. The sequence of siRNA followed our previous research results [18]. Alizarin red staining showed that knockdown Klf10 improved the extracellular matrix calcification of OA chondrocytes (Fig. 2A, B). Likewise, ALP activity was increased in the TBHP-treated group and was significantly decreased with transfection of klf10 siRNA (Fig. 2C). Calcification-promoting gene expressions such as Mmp13, Runx2, Osteopontin, and Osteocalcin increased, while that calcification-inhibiting gene expressions such as MGP and GRP decreased after TBHP treatment (Fig. 2D, E), while Klf10 knockdown reversed these trends. Taken together, these results suggested that knockdown Klf10 attenuated TBHP induced chondrocyte extracellular matrix calcification.

### Klf10 downregulation affects calcium ion content but not phosphate ion

**Fig. 3:**
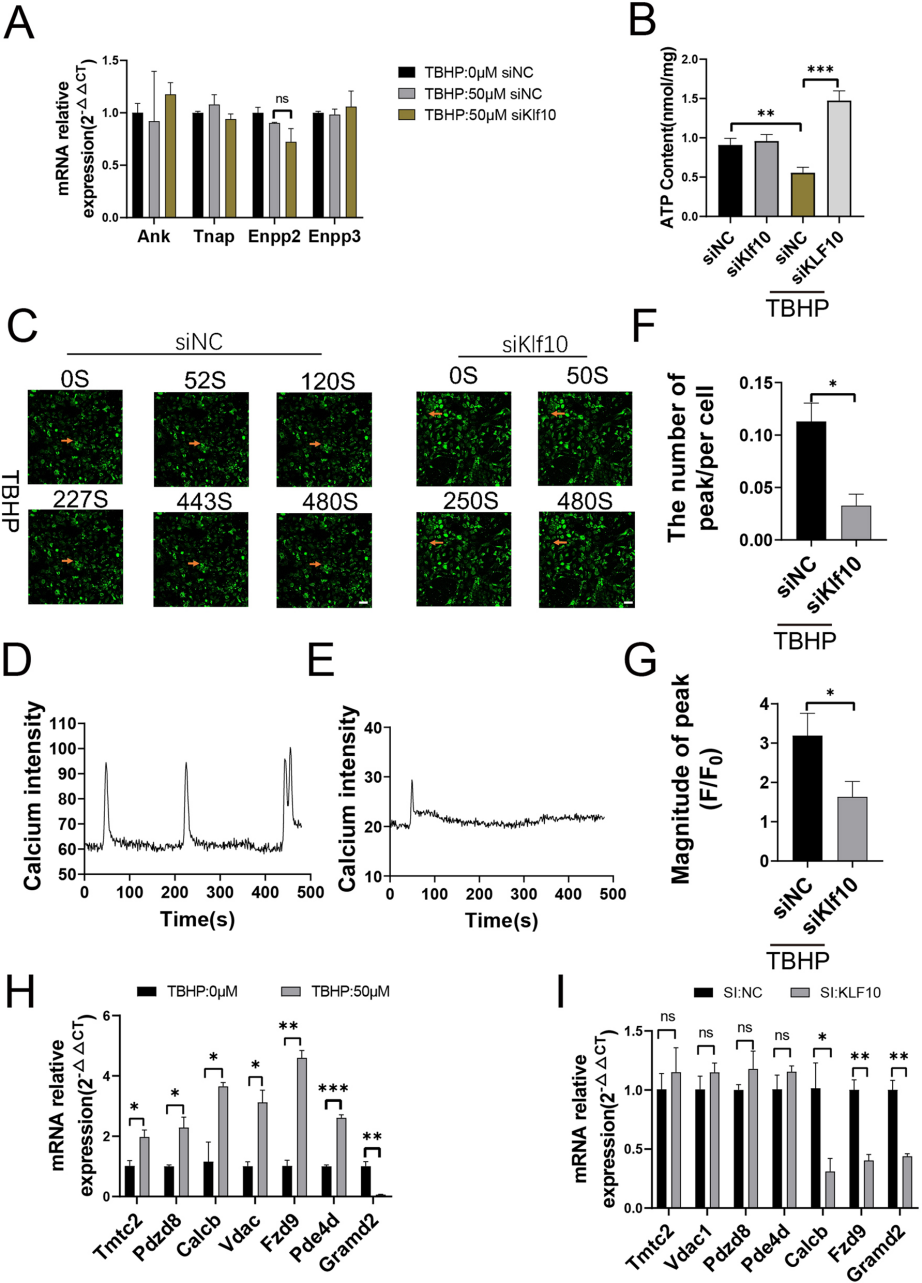
Klf10 downregulation affects calcium ion content but not phosphate ion. **A** Q-PCR was used to detect the expression of related genes (n = 3). **B** The ATP test kit is used to detect ATP produced by articular cartilage cells (n = 3). **C**–**G** Confocal microscopy of cells treated with Fluo-8 dye. The red arrows are positive cells. Data analysis using Zeiss software ZEN (n = 3). Scale bar:100 μm. **H**, **I** Relative gene mRNA expressions of murine chondrocytes were detected using RT-qPCR (n = 3)

The main component of the calcified nodules is hydroxyapatite, which consists of a large number of phosphate ions and calcium ions chelated together [20]. Therefore, to explore the mechanism by which Klf10 affects chondrocyte extracellular matrix calcification, we examined the effect of Klf10 on two major components of hydroxyapatite. The effects of Klf10 on key enzymes regulating PPi production, such as ANK, TNAP, Enpp2, and Enpp3, were evaluated by qPCR. Klf10 had no effect on the gene expression of these key enzymes (Fig. 3A). ATP is a main source of PPi, and when ATP content is consumed in the cell, large amounts of PPi are produced formed [21]. Downregulation of Klf10 led to an increase in ATP (Fig. 3B). Fluo-8 dye showed that calcium ion content was increased after Klf10 downregulation (Fig. 3C–E), and the number of peaks triggered by calcium ion into the cell and the height of peaks were increased (Fig. 3F, G). ChIP sequencing revealed that Klf10 was associated with genes involved in calcium homeostasis (Additional file 1: Fig. S1). We further verified the expression of these calcium homeostasis-related genes at the mRNA level after TBHP treatment and intervention of Klf10 expression. The results showed that except Gramd2, the expression levels of all genes were upregulated with TBHP treatment. While the expressions of Fzd9 and Calcb were attenuated with transfection of Klf10 siRNA (Fig. 3H, I).

### Klf10 regulates Fzd9 to improve chondrocyte extracellular matrix calcification induced by TBHP

**Fig. 4:**
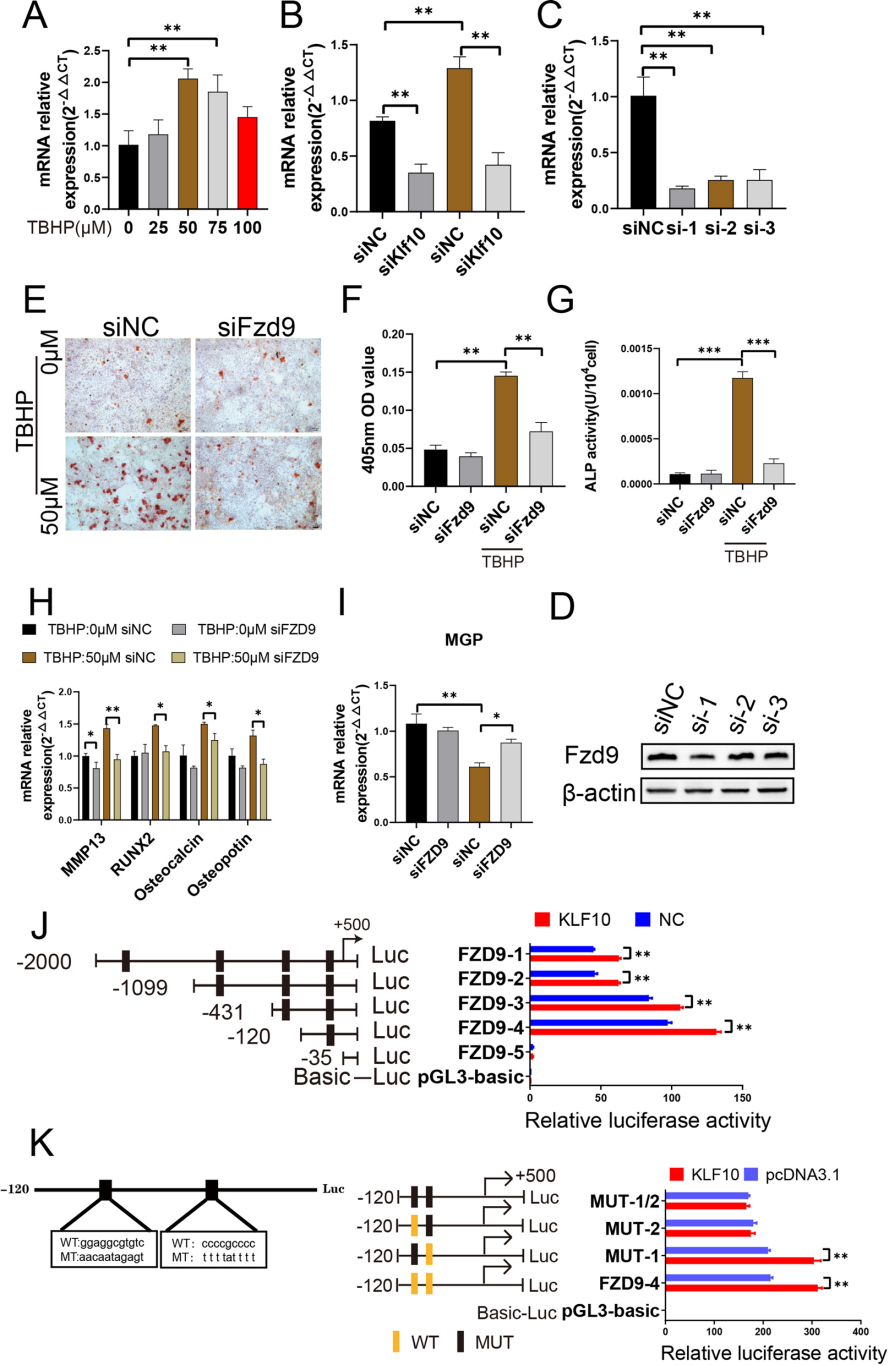
Klf10 regulates Fzd9 to improve chondrocyte extracellular matrix calcification induced by TBHP. **A**–**C** Q-PCR was used to detect the expression of Fzd9 (n = 3). **D** Mouse articular chondrocytes were treated with siRNA (Fzd9) for 48 h. The expressions of Fzd9 was detected by western blot. **E**, **F** Alizarin red dye is used to stain calcium nodules formed by treated chondrocytes (n = 3). Scale bar: 100 μm. **G** The alkaline phosphatase kit was used to measure the alkaline phosphatase activity of chondrocytes (n = 3). **H**, **I** Relative calcification related gene mRNA expressions of murine chondrocytes were detected using RT-qPCR (n = 3). **J** Serially truncated and mutated Fzd9 promoter constructs were cloned and transfected into cells. The relative luciferase activities were determined after Klf10 overexpression. **K** Selective mutation (left panel) analyses identified Klf10-responsive regions in the Fzd9 promoter (right panel)

As Fzd9 is a key gene involved in the regulation of bone mineralization, we tested the role of Fzd9 and Klf10 in regulating calcification. We first verified the expression of FZD9 after treatment with different TBHP dosages. The expression level of FZD9 was upregulated with the increase of TBHP concentrations, and it downregulated with transfection of Klf10 siRNA (Fig. 4A, B). To determine whether FZD9 is associated with chondrocyte extracellular matrix calcification, Fzd9-siRNA was also applied to knock-down of Fzd9 expression. Three Fzd9 siRNA sequences were designed. SiFzd9 #1 displayed the strongest knockdown effect at the mRNA and protein levels in chondrocytes (Fig. 4C, D) and was thus chosen for subsequent experiments. Alizarin red staining showed that Fzd9 knockdown improved the extracellular matrix calcification of OA chondrocytes (Fig. 4E, F). Likewise, ALP activity was increased in TBHP-treated groups and was significantly decreased upon Fzd9 knockdown (Fig. 4G). We overexpressed Klf10 in chondrocytes and performed luciferase assay. The results indicated that the regulatory region may be located between − 120 and − 35 bp (Fig. 4H). Two potential binding sites were present in this region, and single- or double-site mutant promoters were constructed (Fig. 4L). No significant differences in the effects of the wide-type and site-1 mutant sequences were observed; however, site-2 mutation was sufficient to block binding activity (Fig. 4L). Overall, these results indicated that Klf10 regulated Fzd9 expression in chondrocytes. And Klf10 might regulate chondrocyte extracellular matrix calcification via Fzd9 in chondrocytes.

### Restoration of Fzd9 expression aggravates chondrocyte extracellular matrix calcification, and recovery of calcification aggravates chondrocyte senescence

**Fig. 5:**
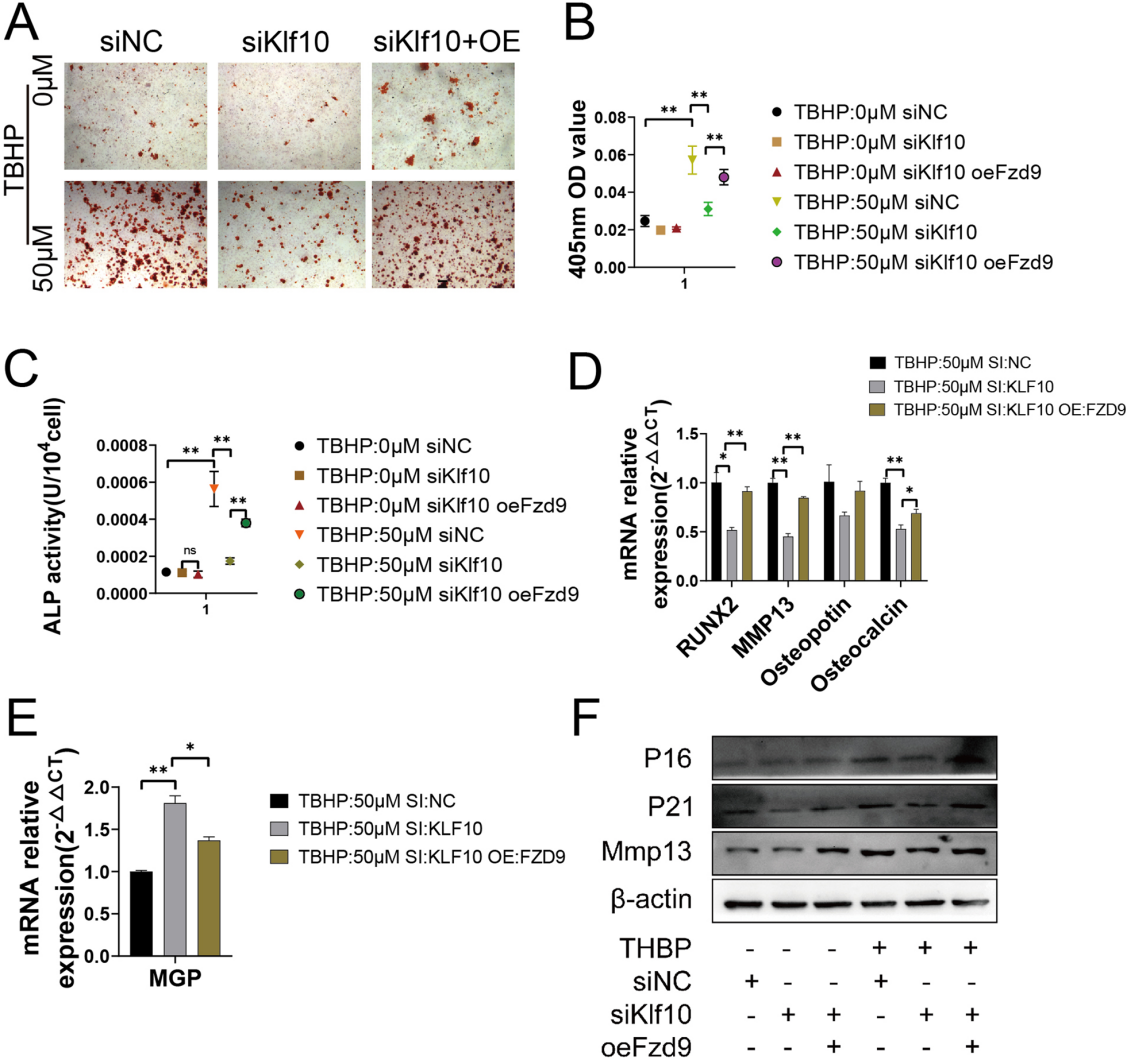
Restoration of FZD9 expression aggravates chondrocyte extracellular matrix calcification, and recovery of calcification aggravates chondrocyte senescence. **A**, **B** Alizarin red dye shows the ability of overexpression treated chondrocytes to form calcified nodules (n = 3). Scale bar: 100 μm. **C** The alkaline phosphatase kit was used to measure the alkaline phosphatase activity of chondrocytes (n = 3). **D**, **E** Relative calcification related gene mRNA expressions of murine chondrocytes were detected using RT-qPCR (n = 3). **F** Murine articular chondrocytes were treated with lentivirus. The expressions of P16, P21 and Mmp13 were detected by western blot

**Fig. 6:**
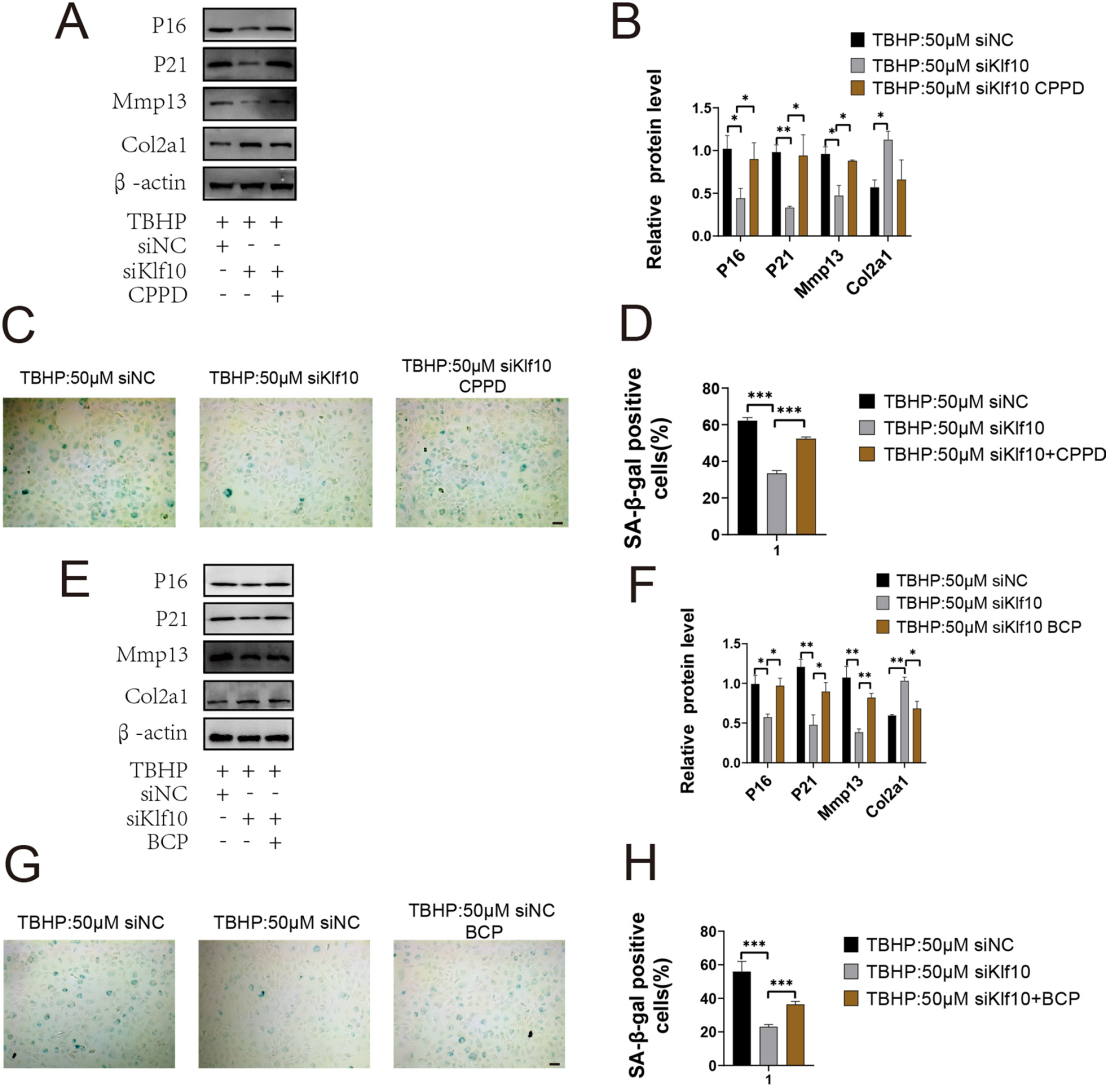
Restoration of Fzd9 expression aggravates chondrocyte extracellular matrix calcification, and recovery of calcification aggravates chondrocyte senescence. **A**, **B** Murine articular chondrocytes were treated with CPPD. The expressions of P16, P21, Mmp13 and Col2a1 were detected by western blot (n = 3). **C**, **D**, **G**, **H** β-Galactosidase staining was used to show senescent chondrocytes, and image J was used to calculate proportions (n = 3). Scale bar: 100 μm. **E**, **F** Murine articular chondrocytes were treated with BCP. The expressions of P16, P21, Mmp13 and Col2a1 were detected by western blot (n = 3)

To further verify the effect of Fzd9 on chondrocyte extracellular matrix calcification, we transfected lentivirus to chondrocytes to overexpress fzd9. Alizarin red staining showed an increase in calcified nodules and recovery of ALP activity after overexpression of Fzd9 (Fig. 5A–C). With regard to effects on related genes, the qPCR results showed that after the restoration of Fzd9 expression, the expression changes of these genes were as expected (Fig. 5D, E). Our previous studies had shown that downregulation of KLF10 could alleviate chondrocyte senescence, but the mechanism was unclear. In order to explore the relationship between chondrocyte extracellular matrix calcification and senescence, the expression levels of senescence markers p16 and p21 were detected by western blotting. The results showed that p16 and p21 expression were decreased by Klf10 downregulation, but were restored by overexpression of FZD9 (Fig. 5F). To further investigate Klf10 regulate senescence by improving calcification, we test the effect of BCP and CPPD, the two main forms of hydroxyapatite. Western blotting and β-galactosidase staining showed that BCP and CPPD induced chondrocyte senescence, however, the effect of CPPD was more obvious. Under CPPD intervention, the expression of p16, p21 and the number of galactosidase-positive cells (Fig. 6A–D) were significantly higher than those with BCP treatment group (Fig. 6E–H). Both BCP and CPPD treatment increased the expression of Mmp13 and downregulated the expression of Col2a1.

### Klf10 downregulation inhibits chondrocyte extracellular matrix calcification and senescence, attenuating articular cartilage degeneration in DMM mice

**Fig. 7:**
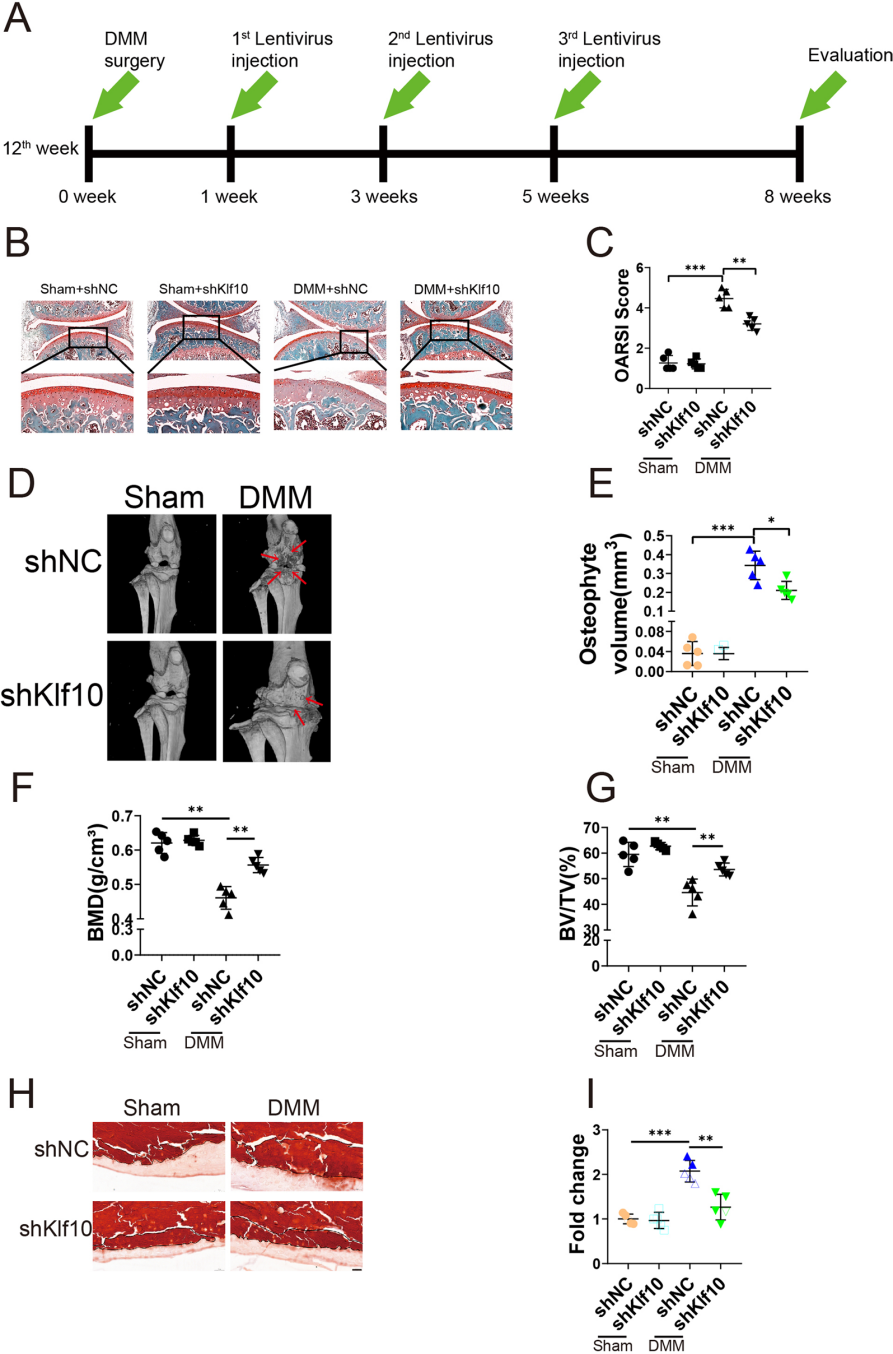
Klf10 downregulation inhibits chondrocyte extracellular matrix calcification and senescence, attenuating articular cartilage degeneration in DMM mice. **A** Schematic of mouse DMM model construction and lentivirus intra-articular injection. **B**, **C** Representative images of Safranin O/Fast Green staining of mouse knee cartilage. The degeneration of articular cartilage in different treatment groups was evaluated using the OARSI score. Scale bar: 100 μm, 50 μm. **D**–**G** Micro-CT was used to detect the knee joints of mice after DMM or sham, and SKY Scan software was used to analyze osteophyte volume, bone density and bone volume fraction (n = 5). **H**, **I** Alizarin red was used to stain the knee sections without decalcification (n = 5)

**Fig. 8:**
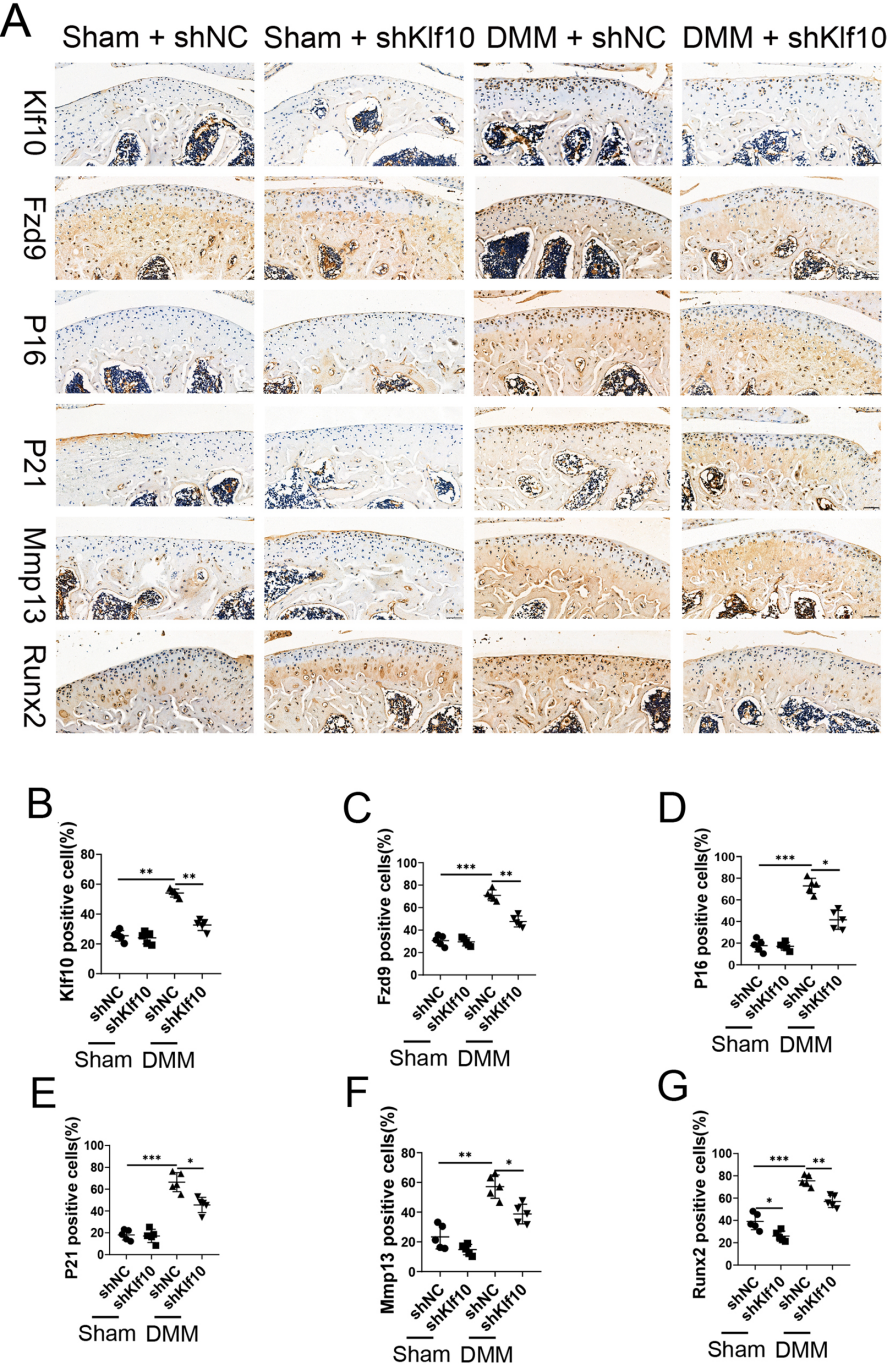
Klf10 downregulation inhibits chondrocyte extracellular matrix calcification and senescence, attenuating articular cartilage degeneration in DMM mice. **A**–**G** Immunohistochemical analysis of the expression of Klf10, Fzd9, Runx2, Mmp13, p21, and p16 in different treatment groups. Scale bar = 20 μm, n = 5

The DMM mouse model was established using 12-week-old mice (Fig. 7A). ShKlf10 lentivirus was injected intraarticularly at 1, 3, or 5 weeks after DMM surgery, and the evaluation was performed 8 weeks after surgery. According to Safranin O/Fast Green staining, the control group showed intact and smooth articular cartilage, while the DMM group developed moderate or severe cartilage degradation. However, inhibiting Klf10 intraarticularly significantly alleviated cartilage destruction (Fig. 7B). The degree of cartilage degradation was scored using OARSI criteria (Fig. 7C). Micro-CT analysis showed that the decrease in bone density and bone volume fraction and the increase in osteophyte volume in the knee area caused by DMM surgery was reversed after downregulation of Klf10 (Fig. 7D–G). Alizarin red staining confirmed that the calcified area of cartilage decreased after Klf10 knockdown (Fig. 7G, H). The expression of senescence markers p16 and p21 was upregulated in the DMM group, and it was decreased upon Klf10 knockdown (Fig. 8A, D, E). In the DMM group, Runx2 and Mmp13 were upregulated, but the expressions were reversed when Klf10 was knocked down (Fig. 6F, G). These results suggested that knock down Klf10 in DMM mice inhibited chondrocyte extracellular matrix calcification and senescence ameliorating articular cartilage degeneration.

## Discussion

Pathological cartilage calcification occurs in almost osteoarthritic joints, however, there is a lack of response that can effectively mitigate the progression of OA [22]. We presented evidences that downregulating Klf10 significantly reduced the formation of calcium-containing crystals in chondrocytes and OA mice, thereby confirming the therapeutic potential of Klf10 as a target for improving crystal-associated OA. The alizarin red staining showed that downregulated Klf10 inhibited chondrocyte calcification by up to 50% in vitro. The inhibitory effect of downregulation of Klf10 on crystal formation was demonstrated in vivo in DMM model. Injection with Klf10 lentivirus in the articular cavity reduced the volume of newly formed calcified deposits after DMM, reducing the degradation of cartilage and proteoglycan loss. In addition, we determined that cartilage calcification promoted chondrocyte senescence.

Pathological cartilage calcification is a hallmark of OA [20]. Calcification can be observed on the surface and in deep layers of the cartilage. Cartilage calcification appears to be an early, age-independent event in the pathogenesis of hip and knee OA [23, 24]. The digital contact radiography (DCR) of patients with OA of the knee or hip undergoing total knee or total hip replacement showed calcification in all cartilage specimens [6]. Understanding the mechanism of chondrocyte extracellular matrix calcification will be a new novel option to determine to delay the progression of OA.

Klf10 has been showed to be played an important role in the musculoskeletal system. Murong You et al. reported that Klf10 knockdown in MC3TC-E1 cells reduced the rate of mineralized nodules formed by the cells [25]. Hawse et al. used Klf10 knockout mice and found that the femur and tibia of 2-month-old female Klf10(−/−) mice showed significant reductions in total bone mineral content, density, and area [26]. Yadav et al. demonstrated that Klf10 is an osteogenic transcription factor that is transcribed in developing bone in a BMP signal-dependent manner [27]. In addition to osteogenic differentiation in osteoblasts, Klf10 also plays a role in osteoclasts [28, 29], myocytes [30, 31], and periodontal membrane cells [32]. Crucially, Klf10, as a direct transcriptional target of the BMP signaling pathway, may be involved in the regulation of endochondral ossification under the synergistic effects of Bmp signaling and a variety of signaling pathways [33]. We found that under TBHP treatment, calcium content in chondrocytes was considerable, and the signal produced by the Fluo-8 dye was increased and enhanced. This trend was reversed when Klf10 was lowered and calcification was alleviated. Therefore, we hypothesized that extracellular matrix calcification induced by Klf10 was related to calcium content. Through ChIP sequencing, we identified the genes involved in calcium ion homeostasis. After TBHP gradient treatment and Klf10 downregulation, only Fzd9 and Calcb were correspondingly expressed. However, in addition to its association with Klf10, Fzd9 also has the function of positively regulating bone mineralization, which qualifies it for further examination. Finally, we demonstrated that Klf10 regulated the transcriptional activity of Fzd9 using a dual luciferase reporter system. Between − 120 and − 35 bp of the Fzd9 promoter, there was a GC box sequence (ccccgcccc) binding to Klf10.

We determined that Klf10 knockdown could improve the extracellular matrix calcification of chondrocytes, which was related to the previous research results that Klf10 knockdown could ameliorate chondrocyte senescence or not? The role of calcium crystals in promoting OA progression was well established [34]. BCP crystals were closely related to chondrocyte hypertrophy [35]. After stimulation of BCP crystals, the expression of markers such as type X collagen and Mmp13 was up-regulated [36]. The degradation of articular cartilage glycoprotein by Mmp13 was also associated with cellular senescence as well as changes in chondrocyte function [37, 38]. CPP crystals stimulated healthy chondrocytes increasing the expression of senescence genes [39]. The two types of calcium crystals coexisted in OA. Therefore, we separately treated chondrocytes with them to observe the effects on chondrocyte senescence. The results showed that both increased chondrocyte senescence, but the treatment of CPP crystals made the senescence worse.

Considering how increasing calcium content causes extracellular matrix calcification, we speculated that it is related to the occurrence of apoptosis. Kim et al. demonstrated that colon cancer cell line HCT-8 was induced apoptosis due to increasing calcium content [40]. Calcium influx mediated by Orai1 channel induces T cell apoptosis [41]. Chondrocyte apoptosis is associated with the release of apoptotic corpuscles, which are considered specialized stromal vesicles involved in pathological mineral deposition [42, 43]. Stromal vesicles are membrane-bound organelles that exert ENPP1 and TNAP activities, produce Pi, are able to concentrate Ca^2+^, and play an initiating role in normal and pathological stromal calcification in many tissues [44, 45]. Moreover, angiogenesis and the lack of phagocytes in articular cartilage led to the accumulation of apoptotic bodies in the extracellular matrix of chondrocytes, which release calcifying precursors that are deposited on collagen fibers, leading to their conversion into calcium crystals. In human and mouse OA, increased chondrocyte apoptosis was detected in OA cartilage, compared to healthy cartilage [46, 47], and apoptosis was positively correlated with the degree of cartilage injury [47–50]. Obviously, cells have a certain self-protection ability, and under large amounts of calcium influx, they may choose to absorb and transform in the form of apoptotic bodies to avoid triggering more signal disorders.

Our research still has shortcomings. We only demonstrated this effect in the TBHP-induced model, and further confirmation is needed to determine whether the effect is the same in other senescence models. Given the important role of Klf10 in regulating chondrocyte extracellular matrix calcification and ameliorating chondrocyte senescence, how increasing calcium content affects calcification needs to be further investigated. Lentiviral transfection is not safe or precise enough to deliver interventions. It is easy to miss the target or interfere with other neighboring tissues, causing unpredictable effects. Fortunately, our immunohistochemical experiments revealed that we did achieve transfection and successfully down-regulated the expression of the relevant molecules in the chondrocytes. If experimental conditions permit, a safer delivery method can be considered.

## Materials and methods

### Primary murine articular chondrocyte culture

Primary murine articular chondrocytes were extracted as previously described [51]. Briefly, C57BL/6 suckling mice aged 7–10 days were sacrificed. Hyaline cartilage of the mouse knee joint was collected. The collected cartilage pieces were digested in 3 mg/mL collagenase D solution for 1 h at 37 °C and 5% carbon dioxide. This was repeated for a second digestion. Thereafter, the cartilage pieces were digested in 0.5 mg/mL collagenase D solution for 12 h. The digested cells were collected by filtration using a 70-micron mesh and were grown in cell culture dishes using a medium containing 10% FBS and 1% penicillin/streptomycin antibody. In total, we extracted cartilage cells from the knee joint of bilateral hind limbs of 40 C57 lactating mice. Up to the end of the whole article, we used the extracted cartilage cells from about 30 lactating mice.

### Cell treatment and siRNA transfection

To establish an in vitro OA chondrocyte model, we referred to our previous results [52] and treated chondrocytes with 50 µM *tert*-butyl hydroperoxide (TBHP, Sigma-Aldrich, USA). Calcification induction of chondrocytes was performed for reference, using a medium containing vitamin C and phosphatase inhibitors. The transfection reagent siRNA-Mate was used to assist the transfection of siRNA, according to the manufacturer’s instructions, and siRNAs for Klf10, Fzd9, and their negative controls were designed and constructed by Gene Pharma (China). The sequences of siRNAs are listed in Additional file 1: Table S1. To verify the effects of Fzd9 and Klf10 knockdown on OA chondrocytes, chondrocytes were treated with 50 µM TBHP after transfection with siFzd9 or siKlf10 treatment for 24 h, and they were then cultured for 24 h.

### Protein extraction and western blotting

The cells were lysed with a lysate containing the protease inhibitor PMSF. The cracking liquid was centrifuged at 17,000×*g* for 5 min. Protein concentrations were measured using a BCA protein detection kit. Equal amounts of protein were used for SDS-PAGE and were then transferred to a PVDF membrane. After blocking in 5% skimmed milk, the membranes were incubated with primary antibodies against Klf10, P16, P21, Fzd9, or β-actin overnight at 4 °C, followed by incubation with the respective horseradish peroxidase (HRP)-conjugated secondary antibodies (goat anti-rabbit or goat anti-mouse; Thermo Fisher Scientific, USA) for 1 h at room temperature. Images were collected using an Invitrogen iBright FL1500 western blot detection system (Thermo Fisher Scientific) with ECL reagents (Solarbio, China).

### Total RNA extraction and real-time PCR analysis

Total RNA was extracted using the Total RNA Isolation Kit (Omega Bio-Tek, Georgia, USA) following the manufacturer’s instructions. A cDNA synthesis kit (RevertAid First Strand cDNA Synthesis Kit; Thermo Fisher Scientific) was used, and real-time qPCR (Forget-Me-Not™ EvaGreen® qPCR Master Mix; Biotium, USA) was performed, according to the manufacturers’ instructions. The primer sequences are listed in Additional file 1: Table S2. The 2^−ΔΔCt^ method was used to calculate the relative expression levels.

### SA-β gal staining

The SA-β gal activity of chondrocytes was detected using an SA-β-gal staining kit (Solarbio). According to the manufacturer’s instructions, cells were diluted to lower cell density, fixed, and incubated overnight with 1× SA-β-gal staining solution in a 37 °C in a CO_2_-free incubator. The percentage of SA-β-Gal-positive cells in randomly selected fields was recorded and analyzed using an optical microscope (Olympus, Japan).

### Study animals and experimental OA induced by DMM

C57BL/6 male mice used in this experiment were purchased from the Guangdong Provincial Medical Laboratory Animal Center and were maintained under specific pathogen-free conditions in an animal facility of the Fifth Affiliated Hospital of Sun Yat-Sen University. A surgical DMM model of OA was induced as previously reported using 12-week-old male mice [53]. The mice were randomly assigned to four groups as follows: a sham group, a sham + shKlf10 group, a DMM group, and a DMM + shKlf10 group. After the mice were anesthetized, their knees were dissected using microsurgical scissors and an operating microscope, and the medial meniscotibial ligament (MMTL) was transected. In mice of the sham group, the joint capsule was cut, and the joint cavity was opened without transection of the MMTL. Mice were killed 8 weeks after surgery to conduct a micro-CT scan and histological evaluation.

### Micro-CT scan and histology

To evaluate the effects of Klf10 inhibition by shKlf10 in vivo, we performed 3D-CT scans of 20-week-old male mice using a micro-CT scanner (nanoScan, Mediso, Hungary) equipped with computer-controlled vertical and horizontal chamber motion. Briefly, the mice were anesthetized and fixed on the micro-CT for scanning. All mice were anesthetized, and knee tissue was collected for subsequent immunohistochemical examination. The knee joint was fixed with 4% paraformaldehyde for 24 h, decalcified with 10% EDTA for 4 weeks, and dehydrated with 20% sucrose for 24 h. After embedding with paraffin, 5-µm thick sections were produced. The paraffin sections were stained with HE and Safranin O-fast green, and the results were evaluated by OA Research Society International (OARSI) scoring. For immunohistochemistry, antigen retrieval was performed on deparaffinized and rehydrated cartilage sections. Endogenous peroxidase activity was then blocked by incubation with 3% hydrogen peroxide for 10 min at room temperature. Nonspecific antibody binding sites were blocked by incubating in 5% normal goat serum. Sections were incubated overnight with primary antibodies against Klf10, Fzd9, Col2a1, Mmp13, and Runx2. Subsequently, sections were washed thoroughly with PBS and were labeled with HRP-conjugated secondary antibody (goat anti-rabbit or goat anti-mouse; Thermo Fisher Scientific) for 1 h at room temperature. Sections were treated with DAB substrate (Solarbio), and nuclei were counterstained with hematoxylin (Beyotime Biotechnology, China). Images were compiled using a Pannoramic 250 FLASH device (3DHISTECH, Hungary).

### Alizarin red staining

Cells were stained using an Alizarin red staining kit according to the manufacturer’s instructions (Solarib, China). In brief, the cells were fixed with 4% paraformaldehyde for 30 min and were rinsed with PBS. Then, the cells were stained with 0.1% (w/v) Alizarin red for 15 min. The matrix mineralization deposition appeared red, and the stained cells were photographed after washing with deionized water for 15 min. 10% ice acetic acid desorbed alizarin red for 15 min. The samples in each treatment group were added with 100 µL of ammonia and reacted at room temperature for 5 min. OD values were then determined at 405 nm.

### Preparation of BCP

Sterile pyrogen-free BCP crystals were synthesized as previously described [36]. Crystals were suspended in sterile PBS and were dispersed by brief sonication. All crystals were determined to be endotoxin-free (< 0.01 EU/10 mg) according to a Limulus amebocyte cell lysate assay.

### Fluo-8

Fluo-8 was dissolved in DMSO to prepare a Fluo-8 stock solution at a concentration of 2–5 mM. The stock solution was diluted with HBSS buffer to 4–5 µM. Equal volumes of this Fluo-8 working solution were added to the collected cell suspension (1 mL), followed by incubation for 30 min at 37 °C. Thereafter, the mixture was centrifuged, the supernatant was discarded, and the cells were washed three times using PBS, followed by confocal microscopy.

### Determination of alkaline phosphatase (ALP) activity

ALP enzyme activity was tested using an ALP assay kit (Jiancheng Biotech, Nanjing, China). Briefly, the treated cells were washed twice with pre-cooled PBS and were lysed with RIPA buffer (Beyotime). The lysate was incubated at 37 °C for 20 min, and ALP enzymatic activity was assessed using a spectrophotometer at 405 nm. Relative ALP activity was normalized to the protein content which was determined using a BCA assay kit (Beyotime).

### Dual-luciferase reporter system

The plasmids (pGL3-Firefly Renilla containing the Fzd9 sequence and a mutant sequence) were synthesized by Promega (Guangzhou, China). Luciferase activity was detected using a dual luciferase assay kit (Promega) according to the manufacturer’s instructions. All experiments were repeated using independent triplicates.

### Lentiviral transfection

Lentivirus vectors encoding Fzd9 or an empty lentiviral vector were constructed by GeneChem (Shanghai, China), and lentivirus transfection was carried out according to the manufacturer’s instructions. Fzd9 overexpression lentivirus was termed Fzd9-OE, and the negative control was termed NC.

### Statistical analysis

Data were presented as the mean ± SD. Statistical analysis was performed by Student’s t-test or one-way analysis of variance (ANOVA). The data shown were representative results from three or more independent experiments. p < 0.05 was considered statistically significant.

## Conclusions

In conclusion, our findings showed that Klf10 regulated TBHP-induced extracellular matrix calcification in chondrocytes, and alleviated chondrocyte senescence. When Klf10 was downregulated, extracellular matrix calcification and intracellular calcium content were improved. After restoring calcification, the trend of chondrocyte senescence being alleviated was reversed. In vivo experiment, Klf10 knockdown in a DMM OA mouse model inhibited pathological cartilage calcification and ameliorated articular cartilage degeneration. These findings demonstrated that Klf10 played a functional role in chondrocyte extracellular matrix calcification during chondrocytes senescence.

### Supplementary Information

**Additional file 1: Figure S1.** Bubble map of CHIP sequencing. GO analysis of CHIP sequencing data. **Figure S2.** TBHP modeling verification experiment. Β-galactosidase staining (A) and expression of senescence marker P16 and P21 (B) in chondrocytes treated with TBHP at different concentrations. **Table S1.** The siRNA sequence information of Fzd9 and negative control. **Table S2.** The sequence information of primers in the article.

## Data Availability

The data that support the findings of this study are available from the corresponding author on reasonable request.
