# Supplementary material for: Klf10 is involved in extracellular matrix calcification of chondrocytes alleviating chondrocyte senescence
Source: J Transl Med. 2024 Jan 13;22:52. doi: 10.1186/s12967-023-04666-7 (PMC10790269; doi:10.1186/s12967-023-04666-7)
Supplement: Supplementary file 1 — Additional file 1: Figure S1. Bubble map of CHIP sequencing. GO analysis of CHIP sequencing data. Figure S2. TBHP modeling verification experiment. Β-galactosidase staining (A) and expression of senescence marker P16 and P21 (B) in chondrocytes treated with TBHP at different concentrations. Table S1. The siRNA sequence information of Fzd9 and negative control. Table S2. The sequence information of primers in the article. [file 12967_2023_4666_MOESM1_ESM.docx]

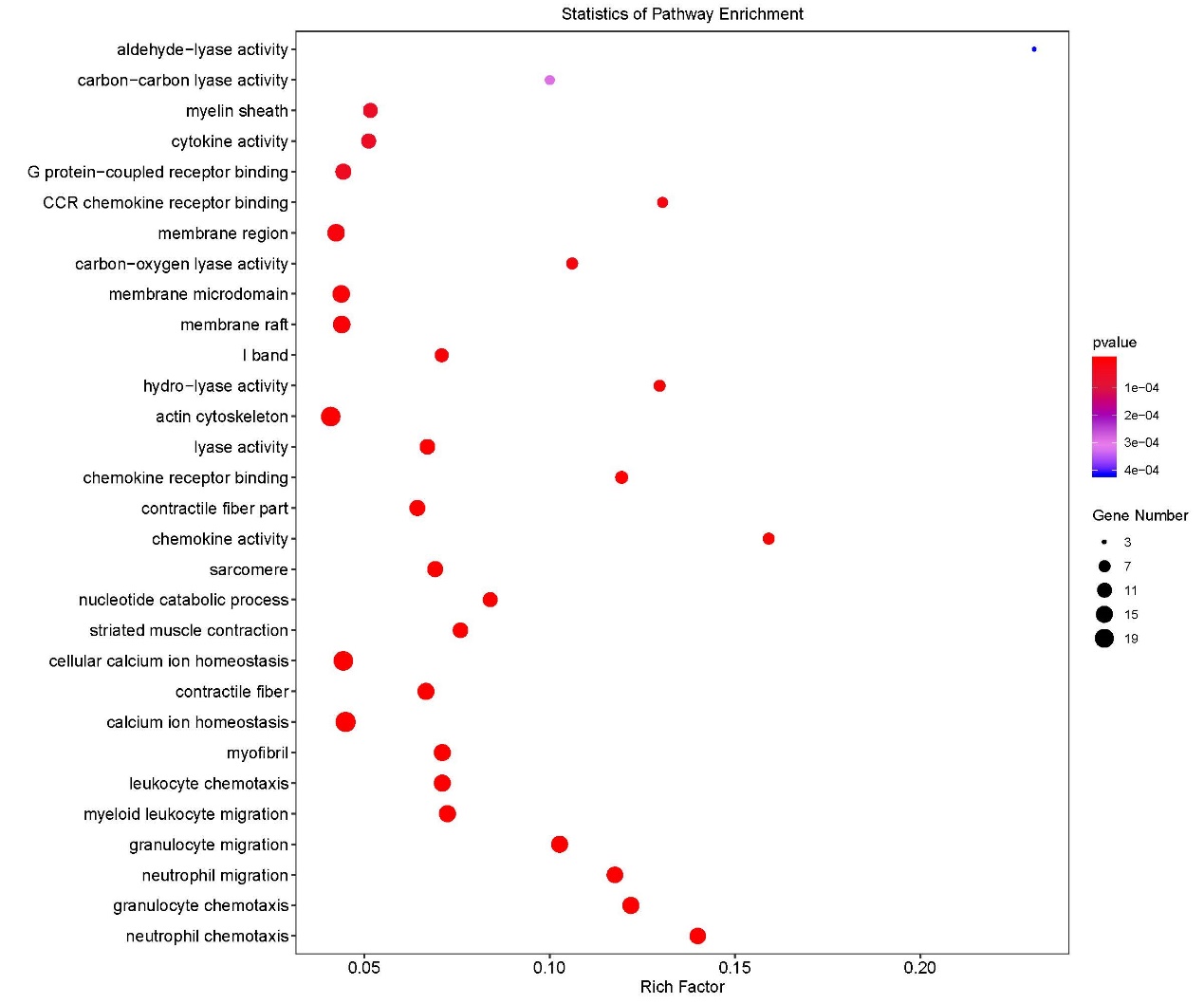


Fig. S1. Bubble map of CHIP sequencing. GO analysis of CHIP sequencing data.


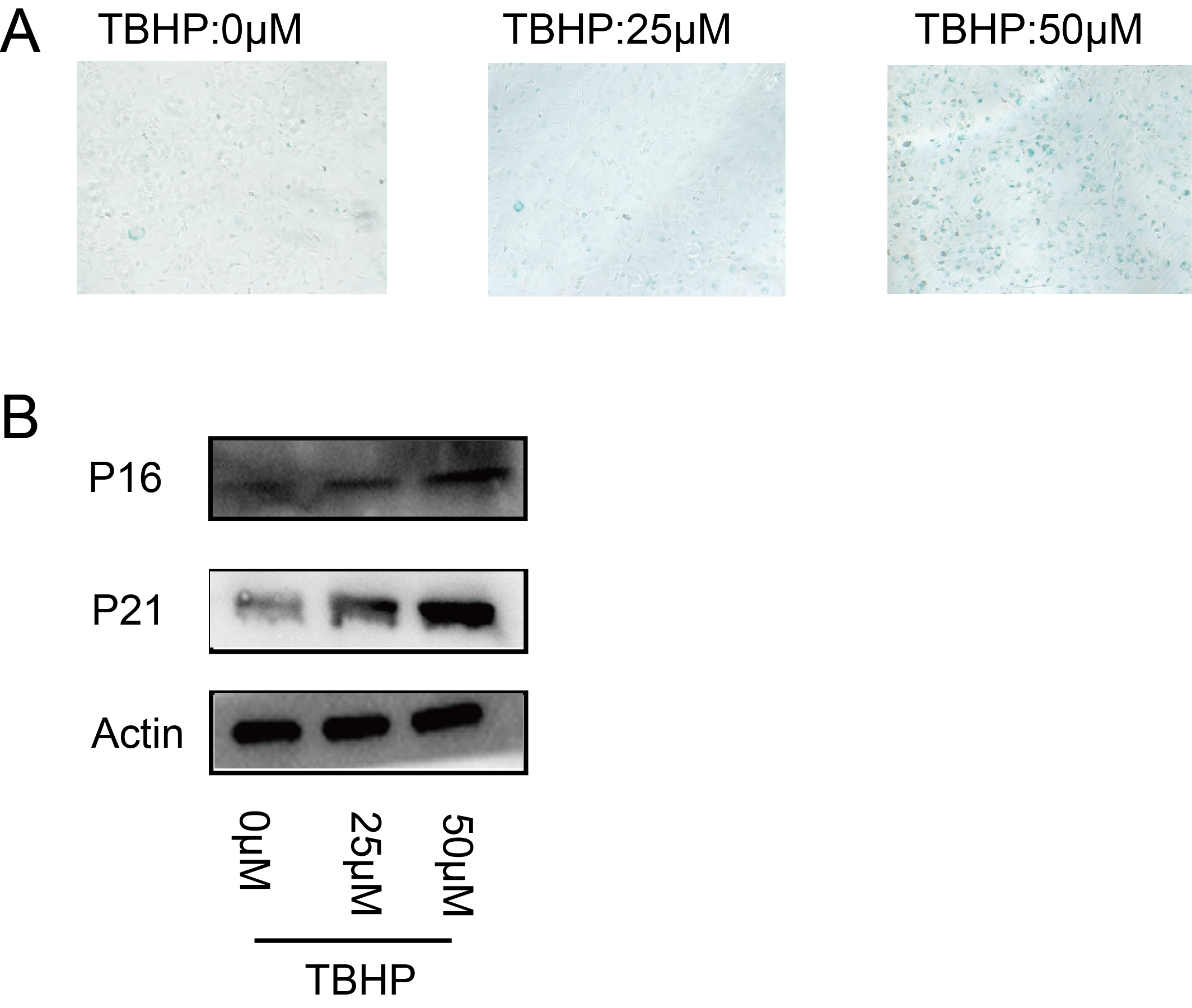
Fig. S2. TBHP modeling verification experiment. Β-galactosidase staining (A) and expression of senescence marker P16 and P21 (B) in chondrocytes treated with TBHP at different concentrations.

| siRNA | Sense (5′-3′) | Antisence (5′-3′) |
| --- | --- | --- |
| Fzd9#1  Fzd9#2  Fzd9#3  Negative control | GGAGAUCGGCCGCUUCGAUTT  GGUCCGCGUUGUGUUUCUUTT  CCUUUCUAUGUGCUACAAUTT  UUCUCCGAACGUGUCACGUTT | AUCGAAGCGGCCGAUCUCCTT  AAGAAACACAACGCGGACCTT  AUUGUAGCACAUAGAAAGGTT  ACGUGACACGUUCGGAGAATT |

Table. S1. The siRNA sequence information of Fzd9 and negative control.

| Gene | Sequence |
| --- | --- |
| Klf10  Fzd9  Mmp13  Runx2  GRP  MGP  Osteocalcin  Osteopontin  Ank  Tnap  Enpp2  Enpp3  Tmtc2  Pdzd8  Calcb  Vdac  Pde4d  Gramd2  β-actin | Forward (5’-3’) ATGCTCAACTTCGGCGCTT  Reverse (5’-3’) CGCTTCCACCGCTTCAAAG  Forward (5’-3’) GTGCCAAGCGATGGAGATCC  Reverse (5’-3’) GCGTAGAGCGAGCAGAAGAA  Forward (5’-3’) CTTCTTCTTGTTGAGCTGGACTC  Reverse (5’-3’) CTGTGGAGGTCACTGTAGACT  Forward (5’-3’) ATGCTTCATTCGCCTCACAAA  Reverse (5’-3’) GCACTCACTGACTCGGTTGG  Forward (5’-3’) CTGTTGGCTCTGGTCCTCTG  Reverse (5’-3’) CATACAGGGACGGGGATTCA  Forward (5’-3’) GGCAACCCTGTGCTACGAAT  Reverse (5’-3’) CCTGGACTCTCTTTTGGGCTTTA  Forward (5’-3’) CTGACCTCACAGATCCCAAGC  Reverse (5’-3’) TGGTCTGATAGCTCGTCACAAG  Forward (5’-3’) AGCAAGAAACTCTTCCAAGCAA  Reverse (5’-3’) GTGAGATTCGTCAGATTCATCCG  Forward (5’-3’) CAGTCAAGGAGGATGCAGTAGA  Reverse (5’-3’) CACTGTAGGCTATCAGGGTGT  Forward (5’-3’) CCAACTCTTTTGTGCCAGAGA  Reverse (5’-3’) GGCTACATTGGTGTTGAGCTTTT  Forward (5’-3’) ATGGCAAGACAAGGCTGTTTC  Reverse (5’-3’) TTGACGCCGATGGCAAAAGT  Forward (5’-3’) CAGTTGACAATGCCTTTGGAATG  Reverse (5’-3’) CACTCTATCACAGGAGGTCTGG  Forward (5’-3’) CCTTGTATCTCAACACCCTGAGT  Reverse (5’-3’) AGTCCCCCAAAAGTCATTGTAGA  Forward (5’-3’) GCAAGTCCGCCTACCTGTTC  Reverse (5’-3’) CACCTCGAAGTCAATCAGCG  Forward (5’-3’) CTCTCAGCACGATATGGGTCC  Reverse (5’-3’) GCAAGAGATGTTTTTCCTGGTCG  Forward (5’-3’) CCCACATACGCCGATCTTGG  Reverse (5’-3’) GTGGTTTCCGTGTTGGCAGA  Forward (5’-3’) TTTTGCCAGTGCAATACATGATG  Reverse (5’-3’) CAGAGCGAGTTCCGAGTTTGT  Forward (5’-3’) TTCCCTCTAGCAACCAACAAATG  Reverse (5’-3’) TTCACCCTTTGAGCCTTCTGA  Forward (5’-3’) GGCTGTATTCCCCTCCATCG  Reverse (5’-3’) CCAGTTGGTAACAATGCCATGT |

Table S2. The sequence information of primers in the article.
